# Virchow-Robin space and aquaporin-4: new insights on an old friend

**DOI:** 10.3325/cmj.2014.55.328

**Published:** 2014-08

**Authors:** Tsutomu Nakada

**Affiliations:** 1 Science Council of Japan, Tokyo, Japan; 2 Center for Integrated Human Brain Science, Brain Research Institute, University of Niigata, Niigata, Japan; 3 Department of Neurology, University of California, Davis, CA, USA

## Abstract

Recent studies have strongly indicated that the classic circulation model of cerebrospinal fluid (CSF) is no longer valid. The production of CSF is not only dependent on the choroid plexus but also on water flux in the peri-capillary (Virchow Robin) space. Historically, CSF flow through the Virchow Robin space is known as interstitial flow, the physiological significance of which is now fully understood. This article briefly reviews the modern concept of CSF physiology and the Virchow-Robin space, in particular its functionalities critical for central nervous system neural activities. Water influx into the Virchow Robin space and, hence, interstitial flow is regulated by aquaporin-4 (AQP-4) localized in the endfeet of astrocytes, connecting the intracellular cytosolic fluid space of astrocytes and the Virchow Robin space. Interstitial flow has a functionality equivalent to systemic lymphatics, on which clearance of β-amyloid is strongly dependent. Autoregulation of brain blood flow serves to maintain a constant inner capillary fluid pressure, allowing fluid pressure of the Virchow Robin space to regulate regional cerebral blood flow (rCBF) based on AQP-4 gating. Excess heat produced by neural activities is effectively removed from the area of activation by increased rCBF by closing AQP-4 channels. This neural flow coupling (NFC) is likely mediated by heat generated proton channels.

Recent studies have strongly indicated that the classic circulation model of cerebrospinal fluid (CSF) is no longer valid (1). The production of CSF is not only dependent on the choroid plexus but also on water flux in the peri-capillary (Virchow Robin) space (2,3). Historically, CSF flow through the Virchow Robin space is known as “interstitial flow” and considered to play a role equivalent to systemic lymphatics. This rather old concept is now fully revived and supported by many studies. One of the most intriguing findings is that clearance of β-amyloid is highly dependent on this flow (4-10).

Several investigations using modern sophisticated technologies revealed that water influx into the Virchow Robin space is controlled by aquaporin-4 (AQP-4), the main subset of the aquaporin water channel family in the brain (11-13). Such activities are shown to be strongly coupled with important physiological phenomena observed in the brain such as neural-flow coupling and sleep (14,15). As a general rule of nature and biological systems, maintenance of Virchow Robin space water hemostasis is critical for apparently distinct, but functionally related biological processes in the brain. This article briefly reviews the modern concept of CSF physiology and the Virchow-Robin space, in particular its functionalities critical for central nervous system neural activities.

## Virchow Robin Space and AQP-4

**Figure 1:**
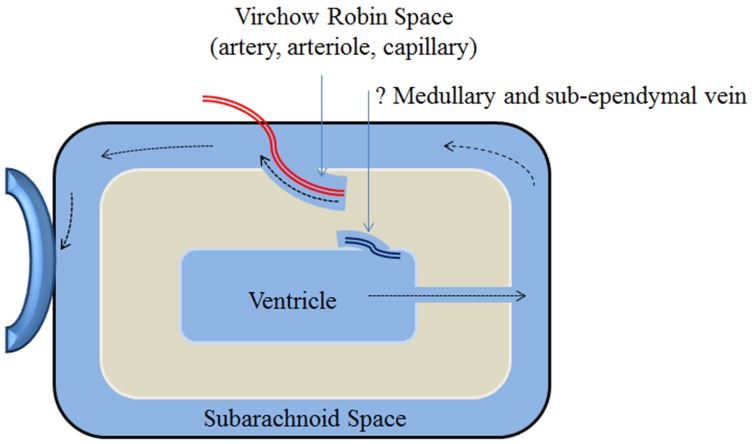
Schematic presentation of the Virchow Robin space and interstitial flow. The ventricles and subarachnoid space represent the cerebrospinal fluid (CSF) space in the brain. The Virchow Robin space is a continuous canal surrounding penetrating vessels. Interstitial flow runs within the Virchow Robin space and drains into the subarachnoid space. Contrary to the classical concept of CSF flow, water CSF within the subarachnoid space is now believed to be dependent on the interstitial flow in the Virchow Robin space. Although not well accepted yet, the Virchow Robin space likely exists surrounding the medullary veins and sub-ependymal veins. As shown in Figure 2, water influx from the systemic circulation into CSF is strongly dependent on the interstitial flow in the Virchow Robin space through aquaporin-4 (AQP-4). Schematic is shown in Figure 4.

Fluid-filled canals surrounding perforating arteries and veins in the parenchyma of the brain were recognized in the early time of modern medicine and referred to as Virchow Robin space based on the first two scientists who described the structure in detail, namely, Rudolph Virchow and Charles Philippe Robin (2,3). It was soon identified that the fluid in the Virchow Robin space may play a role similar to systemic lymphatics (5-7). However, because there is no conventional lymphatic system in the brain the precise nature of the physiological significance of the Virchow Robin space remained to be elucidated. It was initially thought that the fluid in the Virchow Robin space communicates freely with CSF. However, electron microscopic studies subsequently disclosed that the architecture of peri-vascular space is more complex. It is now generally believed (8-10) that peri-capillary space, referred to here as Virchow Robin space, is a continuous space of the CSF system (Figure 1).

The aquaporin family is a large collection of integral membrane proteins that enable the movement of water across biological membranes. Three isoforms, namely AQP-1, AQP-4, and AQP-9, have been identified in mammals in vivo. Expression of AQP-1 within CNS capillaries is actively suppressed, and AQP-1 in the brain is uniquely found in the choroid plexus epithelium. AQP-9 is only scarcely expressed in the CNS and considered to have no significant role (15,16). In contrast, AQP-4 is expressed abundantly in the brain and has a specific distribution: subpial and perivascular endfeet of astrocytes (12,13,15,16). It is now clear that water influx into the Virchow Robin space and, hence, CSF production is highly dependent on AQP-4. Active suppression of AQP-1 expression within brain capillaries is essential for proper maintenance of the blood brain barrier (BBB), preventing excessive movement of water across capillary walls (17,18), a function similar to tight junctions. AQP-4 on perivascular endfeet of astrocytes further regulates water homeostasis in the Virchow Robin space.

## β-amyloid clearance and Alzheimer disease

**Figure 2:**
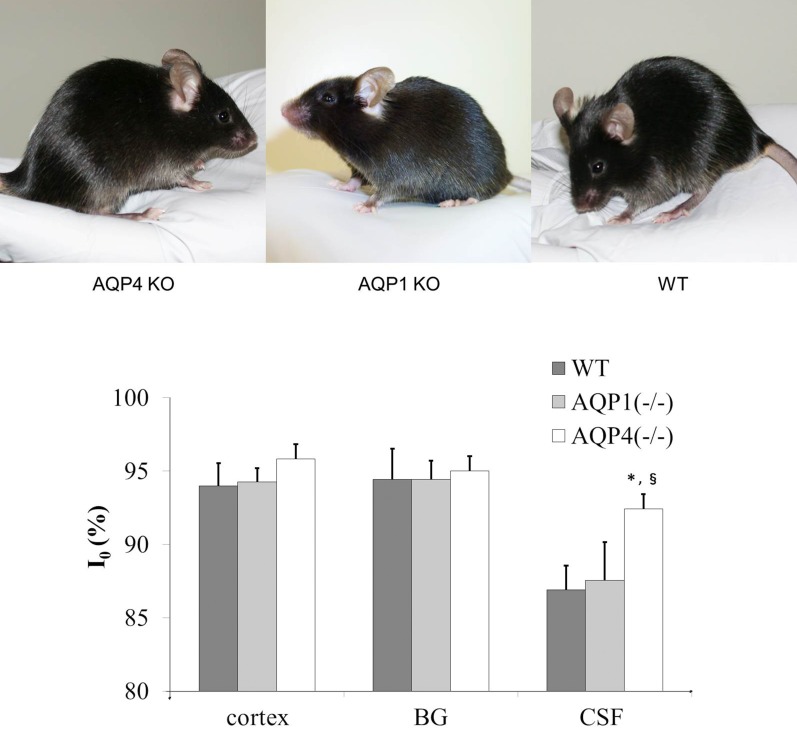
In vivo dynamic study of water influx (13). Detailed description of H_2_O^17^ JJ vicinal coupling proton exchange (JJVCPE) imaging can be found in the Methods. In brief, the technique allowed for tracing water molecules in vivo non-invasively. The figure gives a summary of quantitative analysis of water influx into the region of interest (ROI). I_0_ (see Figure 6 for definition) represents dynamic signal intensity, the decline of which inversely correlates with H_2_O^17^ influx into the region, namely, higher water influx gives lower I_0_ value. Values of I_0_ in cortex and basal ganglia (BG) are virtually identical among the three groups. In contrast, I_0_ of cerebrospinal fluid (CSF) within the third ventricle is significantly higher in AQP-4 knock out (KO) mice compared to AQP-1 KO and wild type (WT) mice. I_0_ of CSF within the third ventricle in AQP-1 KO mice is virtually identical to WT mice. The data indicate that water influx into the CSF is regulated by AQP-4, and not by AQP-1. *P < 0.01 vs WT, ^§^*P* < 0.01 vs AQP1(−/−). WT: wild type, AQP1(−/−): AQP1 knockout, AQP4(−/−): AQP4 knockout mice. Note: WT and KO mice are phenotypically indistinguishable.

Water influx into the CSF system from the blood stream has been shown to be regulated by AQP-4, not AQP-1, findings highly compatible with the Oreskovic and Klarica hypothesis of CSF homeostasis (Figure 2) (13). Thus, brain interstitial flow, the system which is equivalent to systemic lymphatics, also turns out to be regulated by AQP-4.

**Figure 3:**
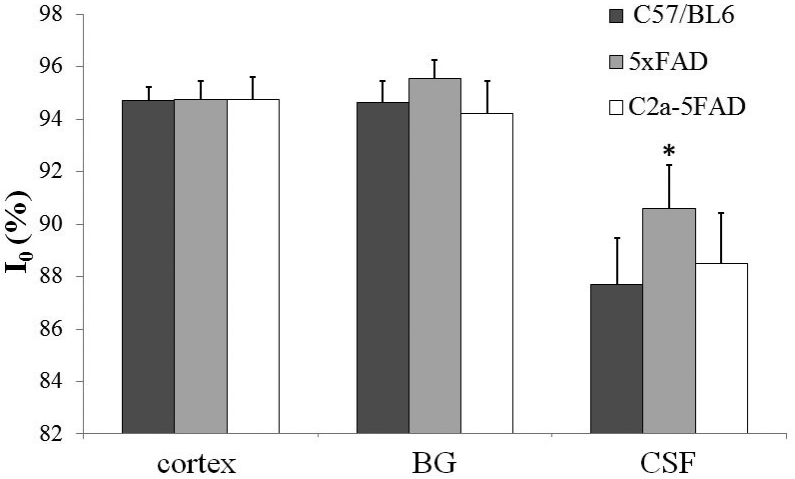
Water influx study in transgenic mice. H_2_O^17^ JJ vicinal coupling proton exchange (JJVCPE) imaging dynamic study showed that only senile plaque bearing transgenic mice (5xFamilial Alzheimer Disease [FAD]) showed a decline in water influx into the cerebrospinal fluid (CSF) system similar to aquaporin-4 (AQP-4) knockout mice. β-amyloid overproducing transgenic mice without senile plaque formation (C2a-5FAD) showed a virtually identical influx with control mice (C57/BL6). The study indicates that disturbance in β-amyloid clearance through the interstitial flow play a critical, if not sole, role in the pathogenesis of Alzheimer disease.

The basic function of lymphatic is drainage of cellular debris subjected to molecular scrutiny before returning to venous circulation. Water influx into the CSF system through the Virchow Robin space is likely to play an essential role in clearing toxic proteins from the brain parenchyma. One of the most intriguing examples is β-amyloid. Interstitial flow is shown to be responsible for β-amyloid clearance (8,9,14). Senile plaque bearing transgenic mice showed significant decline in water influx into the CSF system to the extent similar to AQP-4 knockout mice (19). In contrast, transgenic mice with enhanced production of β-amyloid, but without senile plaque formation, showed normal influx into the CSF through the Virchow Robin space (Figure 3).

β-amyloid is shown to be essential for synaptic formation (20). Nonetheless, excess β-amyloid can result in aggregation of the protein and, in turn, senile plaque formation. Drainage of β-amyloid by interstitial flow through the Virchow Robin space into CSF is likely to be critical for maintaining proper homeostasis of β-amyloid production and clearance. In this matter, the ventricular system plays a role similar to lymph nodes of the systemic lymphatic system for neutralizing potential β-amyloid toxicity. Indeed, prealbumin (also known as transthyretin), the protein abundantly present in CSF, is a chaperon for β-amyloid, and prevents β-amyloid's natural tendency of accumulating into plaques. The delicate balance between β-amyloid production and its clearance appears to be a critical factor for maintaining proper neural function. Disruption in its homeostasis may play an important role in the development of Alzheimer disease. Indeed, positron emission tomography (PET) studies in patients have unequivocally shown this to be the case (19).

## Heat production and neural flow coupling

**Figure 4:**
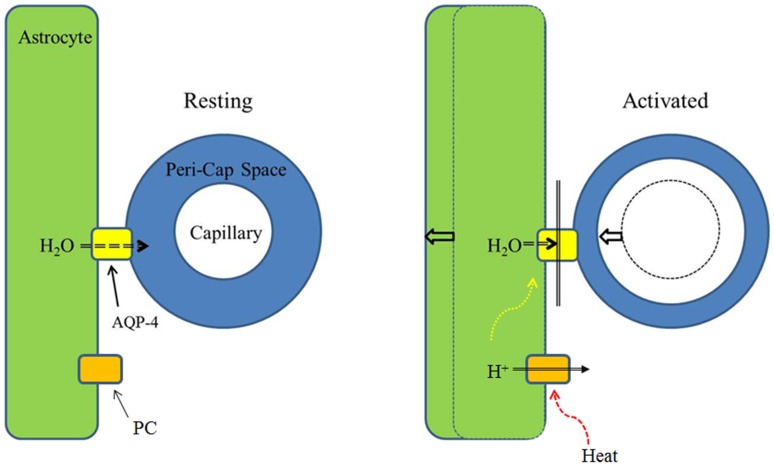
Schematic presentation of the hypothesis. Neural activity is known to produce two distinctive phenomena, namely, astrocyte swelling and increased regional cerebral blood flow (rCBF). Since an increase in rCBF associated with neural activities occurs within an area limited to 250 µm around the site of the neural activity, it is very likely to be a phenomenon associated with capillaries. Inhibition of aquaporin-4 (AQP-4) effectively increased rCBF, supporting the hypothesis presented here. Inhibition of AQP-4 results in blockage of water flow from astrocyte into the Virchow Robin space (Peri-cap space). This in turn results in astrocyte swelling and capillary expansion due to reduction of the peri-capillary Virchow Robin space. The process associated with brain activation can be explained in the same manner. The trigger is likely to be excess heat produced by neural activities, which in turn open heat gated proton channels (PC) similar to those found in leukocyte.

Increased regional cerebral blood flow (rCBF) associated with brain activation is a well-recognized phenomenon known as neural flow coupling (NFC). Since this is a micro, rather than macro environmental event occurring within an area limited to 250 µm around the site of neural activity, the regulatory mechanism for NFC should be within the capillaries (21), vessels devoid of muscle and its neural control. Therefore, it follows that increased blood flow is based on mechanical processes. Since perfusion pressure of the brain is specifically controlled by autoregulation (22), an increase in rCBF should be accomplished by reducing the pressure around capillaries, namely, the pressure within the Virchow Robin space. NFC is accompanied by other phenomena within adjacent astrocytes, namely, astrocyte swelling and transient intracellular alkalization. AQP-4 has been shown to be responsible for astrocyte swelling and dynamic changes in water volume of the peri-capillary space associated with neural activities (12,13). Accordingly, a hypothesis for the underlying mechanism of NFC based on AQP-4 functionality was proposed (Figure 4). Recent studies unambiguously support this new hypothesis. Blocking AQP-4 by an inhibitor effectively increases rCBF (15).

It was once believed that neural-flow coupling serves to ensure adequate neuronal “nutrient supply.” This intuitively appealing notion fails to account for the large quantitative discrepancy between demand and supply. The amount of essential nutrients delivered by the increased rCBF, such as oxygen and glucose, exceeds actual consumption by more than six times. Such a large discrepancy is virtually unknown in any other biological system, indicating that a factor other than nutrient supply requires the observed disproportionate increase in rCBF (23).

**Figure 5:**
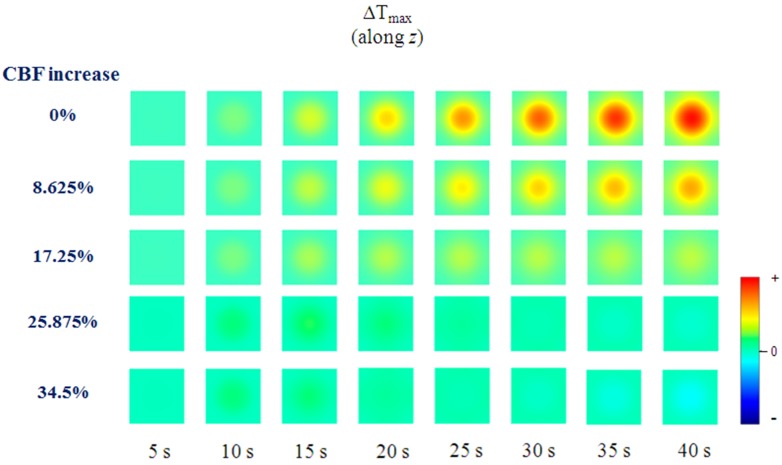
Schematic presentation of temperature changes-associated activation. A detailed description of simulation study can be found in the Methods. The figure shows temperature changes of the activated area (Figure 7) with continuous neural activities in seconds and associated percentage of cerebral blood flow (CBF) increase. Note that with 34.5% increase in CBF, virtually 100% of excess heat can be removed from the cortex.

Information processing by brain generates considerable heat. Water flow is known to play a significant role in heat removal. It is, therefore, highly conceivable that the apparent surfeit in rCBF increase may actually serve as a quick removal system of excess heat generated by neural activities. Simulation studies correlating neural heat generation and rCBF heat removal capacity demonstrated that an rCBF increase of approximately 19 mL/100 g/min, as observed with brain activation, virtually removed 100% of the excess produced heat (Figure 5). The results unequivocally support that excess heat removal is likely the main role of increased rCBF associated with brain activation (24). Neural activities are associated with a transient intracellular alkaline shift and astrocyte swelling (12,25). These events strongly imply that blocking AQP-4 may be initiated by opening a heat gated proton channel as has been shown in leukocytes (26).

## Conclusion

The Virchow Robin space is a peri-capillary fluid filled space responsible for interstitial flow in the brain. Water influx into the Virchow Robin space and, hence, interstitial flow is regulated by aquaporin-4 (AQP-4) localized in the endfeet of astrocytes, connecting the intracellular cytosolic fluid space of astrocytes and the Virchow Robin space. Interstitial flow has a functionality equivalent to systemic lymphatics, and on which clearance of β-amyloid is strongly dependent.

Autoregulation of brain blood flow serves to maintain a constant inner capillary fluid pressure, allowing fluid pressure of the Virchow Robin space to regulate regional cerebral blood flow (rCBF) based on AQP-4 gating. Excess heat produced by neural activities is effectively removed from the area of activation by increased rCBF by closing AQP-4 channels. This neural flow coupling (NFC) is likely mediated by heat generated proton channels.

## Methods

### H_2_O^17^ JJ vicinal coupling proton exchange (JJVCPE) imaging

*Concept.* ^17^O and an adjacent proton will exhibit JJ vicinal coupling. In water, the protons of the water molecule and ionized proton of the dissolved molecule can exchange between each other. Accordingly, appropriately designed ^17^O labeled molecules can alter the apparent T2 of water molecules under nuclear magnetic resonance (NMR) experiments. Using T2 weighted imaging, this property can be developed into imaging that is capable of quantifying the contents of the target molecules, akin to radioactive tracer imaging such as positron emission tomography (PET). This imaging technique is referred here to as JJ vicinal coupling proton exchange (JJVCPE) imaging (27,28).

Signal intensity change, δS, of the voxel with ^17^O labeled substrate can be given by:

(equation 1) where S_0_ is original signal intensity, TE, echo time, ρ, relative concentration of ^17^O labeled substrate, and τ, proton exchange rate. Although it is difficult to obtain the absolute concentration of the ^17^O labeled target molecule with this imaging method, it is still possible to obtain dynamic data for a target molecule in space and time.

*Animal preparation.* Mice breathing spontaneously and anesthetized with an intra-peritoneal administration of urethane (1.2 g/kg), were positioned on their backs in a custom made Plexiglas stereotaxic holder. The mouse head was fixed in position by ear and tooth bars. Rectal temperature was maintained at 37şC ± 0.5 using a custom designed temperature control system. Oxygen saturation (SpO2) was monitored throughout the MR study using a pulse oxymeter, Mouse Ox (STARR Life Sciences Co, Oakmont, PA, USA) with probe placement on the left thigh. Data from animals showing SpO2 of less than 93% at any point in the experiment were discarded. (One KO mouse and one KO mouse). 0.2 mL normal saline containing 20% of H_2_^17^O was administered as an intravenous bolus injection at the 75th phase (10 minutes after the first scan) using an automatic injector at 0.04 mL/s through PE10 tubing inserted into the right femoral vein.

*Imaging parameters.* MRI experiments were performed on a 15-cm bore 7 T horizontal magnet (Magnex Scientific, Abingdon, UK) with a Varian Unity-INOVA-300 system (Varian Inc, Palo Alto, CA, USA) equipped with an actively shielded gradient. A custom made one turn surface coil, 20 mm outer diameter, was used for RF transmission. Adiabatic double spin echo prepared using rapid acquisition with refocused echoes (RARE) was utilized with the following parameter settings: single slice (2 mm thick), 128 × 128 matrix image of 20 × 20 mm field of view, TR 2000 ms, Echo Train 32, TE for first echo 8.8ms, Echo Spacing 5 ms, effective TE 84.8 ms. Imaging slabs were set 6 mm caudal from the top of the cerebrum. A total of 525 phases (scan time 70 minutes) were obtained at 8 seconds intervals.

**Figure 6:**
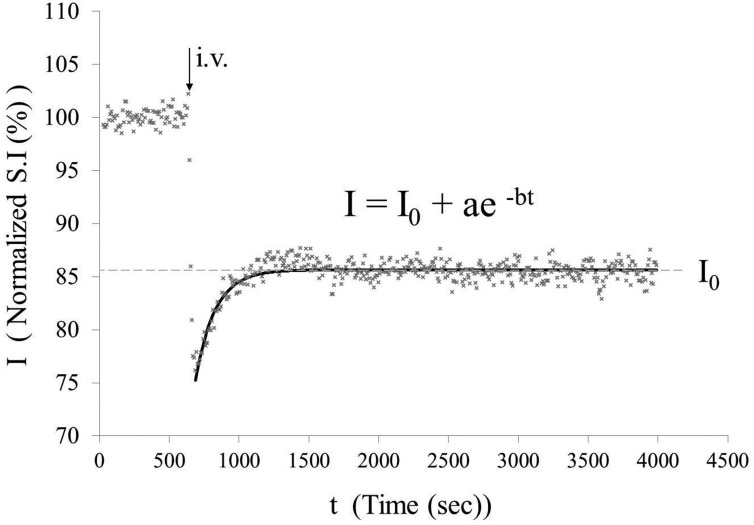
Intensities at the steady state of each area, expressed as percentage against the averaged intensity of identical pixel prior to administration of H_2_O^17^, were determined by fitting their time course by the function: I = I_0_+ae^(-bt)^. I_0_ denotes the normalized signal intensity at infinite time (*t* = ∞) calculated from the fitted curve (13).

*Data analysis.* Images were analyzed by image processing software (MR vision, MRVision Co. Winchester, MA, USA). Averaged percentage of intensities, which reflect relative influx of H_2_^17^O in three areas, namely the cortex, basal ganglia, and third ventricle was plotted against time. Intensities at the steady state of each area, expressed as percent against the averaged intensity of identical pixel prior to administration of H_2_^17^O, were determined by fitting their time course by the function (Figure 6) (equation 2):

Subsequently, numerical data were subjected to *t* test for group analysis. *P* < 0.05 was regarded as statistically significant. All data are shown as mean ± standard deviation.

### Measurement of baseline absolute regional cerebral blood flow by MRI

Baseline absolute regional cerebral blood flow (rCBF) was measured by magnetic resonance imaging (MRI) on a 15-cm bore, 7 T horizontal magnet (Magnex Scientific) using a Varian Unity-INOVA-300 system (Varian Inc.) equipped with an actively shielded gradient. A custom designed eight element, ϕ40mm bird-cage coil was used for RF transmission.

Spontaneously breathing mice were anesthetized using urethane (1.2 g/kg, intraperitoneally). The following anesthesia protocol was used to avoid respiratory depression. Urethane, 600 mg/kg, was administered intraperitoneally at *t* = 0; two-additional doses of urethane, 300 mg/kg, were subsequently administered 10 and 20 minutes after the first dose. Mice were placed on their back in a custom designed Plexiglas stereotactic holder, and the head was immobilized by ear and tooth bars. Rectal temperature was maintained at 37 ± 0.5 şC using a temperature control system. Oxygen saturation (SpO2) was monitored throughout the study procedure using a pulse oxymeter, Mouse Ox (STARR Life Sciences Co), with probe placement on the left thigh.

Cerebral perfusion images were measured using continuous arterial spin labeling (CASL) with centric ordered snapshot-FLASH. Adiabatic inversion for inflowing arterial protons was accomplished with axial gradient of 428.5 Hz/mm and continuous RF transmission of approximately 600 Hz at a frequency offset of ±4285 Hz alternatingly from the imaging slab and mirror plane. This protocol placed the inversion plane at ±10mm from the imaging plane. After a 2-second inversion period, a 500 milliseconds delay was set in order to eliminate the effect of regional transit time delay. Imaging parameters of the pulse sequence were as follows: repetition time/echo time (TR/TE) 4/2 milliseconds; repetition time 5 seconds; field of view 20 × 20 mm; slice thickness 2 mm; matrix size 128 × 64. Sixty-four image pairs were summed to improve signal to noise ratio.

Magnetization transfer was measured with the identical conditions as for the cerebral perfusion measurement but without axial gradient for adiabatic inversion. T1 measurement was accomplished using centric ordered snapshot-FLASH with hyperbolic secant inversion pulse, 64 point inversion delay (100 to 6400 milliseconds) and 10-second repetition time.

Quantitative rCBF maps were calculated from cerebral perfusion images, T1 maps and magnetization transfer (MTR) maps according to the method described by Ewing et al. rCBF maps were computed using an image preparing software (MR Vision, MR Vision Co, Menlo Park, CA, USA) on a Linux workstation (Dell, Round Rock, TX, USA). Region of interest (ROI) approximately 3 × 4 mm was set to the cortical surface.

*AQP knockout mouse preparation*. AQP-1 deficient mice were produced by homologous recombination using an ES cell line from the C57BL/6 strain as follows. We first isolated a genomic fragment carrying exons 0-4 of the AQP-1 gene from C57BL/6 mouse genomic DNA. The 34-bp loxP sequence with 26-bp linker sequence was inserted into the site 210 bp upstream of exon 2. A 1.8 kb DNA fragment, which carried the 34 bp loxP sequence and Pgk-1 promoter-driven neomycin phosphotransferase gene (neo) flanked by two Flp recognition target (frt) sites was then inserted into the site 190 bp downstream of exon 3. The targeting vector ptvAQP-1 or 4-flox contained exon 2-3 of the AQP-1 or 4 gene flanked by loxP sequences 7.7 kb upstream and 2.0 kb downstream genomic sequences, and 4.3 kb pMC1DTpA. ES cells were cultured on mitomycin C-treated neomycin-resistant fibroblasts in Dulbecco's modified Eagle's medium (DMEM, high glucose; Invitrogen, Carlsbad, CA, USA) supplemented with 17.7% ES-cell qualified fetal calf serum (Invitrogen), 88.4 μM non-essential amino acids (Invitrogen), 884 μM sodium pyruvate (Sigma, St. Louis, MO, USA), 88.4 μM 2-mercaptoethanol (Sigma), and 884 U/mL murine leukemia inhibitory factor, ESGRO (Chemicon International, Temecula, CA, USA). Linearized ptvAQP-1-flox was electroporated into ES cells, and G-418 (175 μg/mL)-resistant clones were picked up. Recombinant clones were identified by Southern blot hybridization analysis. Recombinant ES cells were injected into eight-cell stage embryos of the CD-1 mouse strain. The embryos were cultured to blastocysts and transferred to the uterus of pseudopregnant CD-1 mice. The resulting chimeric mice were mated to C57BL/6 mice, and offspring (AQP-1 or 4+/flox) were further crossed with TLCN-Cre mice to yield heterozygous (AQP-1 or 4 +/−) mice. Homozygous AQP-1 or 4-KO mice were obtained by crossing heterozygous pairs.

AQP4-deficient mice were produced by homologous recombination using an ES cell line from the C57BL/6 strain. We first isolated a genomic fragment carrying exons 0-4 of the AQP4 gene from C57BL/6 mouse genomic DNA. A 1.8 kb DNA fragment, which carried the 34 bp loxP sequence and Pgk-1 promoter-driven neomycin phosphotransferase gene (neo) flanked by two Flp recognition target (frt) sites (12,13) was then inserted into the site 100 bp upstream of exon 2. The 34-bp loxP sequence with 26-bp linker sequence was inserted into the site 185 bp downstream of exon 3. The targeting vector ptvAQP4-flox contained exon 2-3 of the AQP4 gene flanked by loxP sequences 4.5 kb upstream and 6.0 kb downstream genomic sequences, and 4.3 kb pMC1DTpA. ES cells were cultured on mitomycin C-treated neomycin-resistant fibroblasts in DMEM (high glucose) (Invitrogen) supplemented with 17.7% ES-cell qualified fetal calf serum (Invitrogen), 88.4 μΜ non-essential amino acids (Invitrogen), 884 μΜ sodium pyruvate (Sigma), 88.4 μΜ 2-mercaptoethanol (Sigma), and 884 U⁄mL murine leukemia inhibitory factor, ESGRO (Chemicon International). Linearized ptvAQP4-flox was electroporated into ES cells, and G-418 (175 μg⁄mL)-resistant clones were picked up. Recombinant clones were identified by Southern blot hybridization analysis. Recombinant ES cells were injected into eight-cell stage embryos of CD-1 mouse strain. The embryos were cultured to blastocysts and transferred to the uterus of pseudopregnant CD-1 mice. The resulting chimeric mice were mated to C57BL⁄6 mice, and offspring (AQP4+/flox) were further crossed with TLCN-Cre mice (12,13) to yield heterozygous (AQP4 +⁄-) mice. Homozygous AQP4-KO mice were obtained by crossing heterozygous pairs.

### Transgenic mouse preparation

*Senile plaque harvested transgenic mouse.* Male B6SJL-Tg (APPSwFlLon, PSEN1*M146L*L286V) 6799Vas/Mmjax mice (5xFAD mouse, one to two months of age) were obtained from Jackson Laboratory (Bar Harbor, ME, USA), and raised in our laboratory until 18 months of age. These transgenic mice overexpress both mutant human APP (695) with the Swedish (K670N, M671L), Florida (I716V), and London (V717I) Familial Alzheimer Disease (FAD) mutations and human PS1 harboring two FAD mutations, M146L and L286V. Expression of both transgenes is regulated by neural-specific elements of the mouse Thy1 promoter to drive overexpression in the brain. Animals were housed in standard housing conditions with a 12-hour light/dark cycle and provided water and food ad libitum.

*Senile plaque negative amyloid-β overexpressed transgenic mouse.* C57BL/6NCrl-NEP^tm2Tsna^ (C2a-5FAD) mice were produced by homologous recombination using the ES cell line RENKA. A genomic fragment of the NEP gene (*Nep*) was isolated from a C57Bl/6 mouse genomic BAC clone (BACPAC Resources Center, Oakland, CA, USA) using the BAC subcloning kit (Gene Bridges GmbH, Heidelberg, Germany). We placed the cDNAs encoding mutant human APP (695) with the Swedish (K670N, M671L), Florida (I716V), and London (V717I) FAD mutations (770 residue isoform numbering) and human PS1 harboring two FAD mutations, M146L and L286V into a single targeting vector. To this end, we used the foot-and-mouth disease virus 2A self-cleaving peptide, which enables efficient polycistronic expression. The 2A sequence (5′-aaa att gtc gct cct gtc aaa caa act ctt aac ttt gat tta ctc aaa ctg gct ggg gat gta gaa agc aat cca ggt cca-3′) was inserted between the APP cDNA and PSI cDNA in frame, the APP cDNA amplified by polymerase chain reaction (PCR) to replace the translation termination codon with 2A. A 34-bp loxP sequence, a 1.2 kb fragment of genomic DNA containing mouse CamkIIα promoter, a 2.2 kb APP cDNA with 2A, a 1.6 kb PS1 cDNA, and a 0.2 kb SV40pA sequence was amplified by PCR and subcloned into Neo vector containing a loxP-FRT-PGK-gb2-neo-FRT cassette (Gene Bridges GmbH). The 5′ arm of 4.2 kb, carrying exons 10-11 of the NEP gene, and the 3′ arm of 7.7 kb, carrying exons 13-15 of the NEP gene, was amplified by PCR. The loxP- CamkIIα promoter-APP-2A-PS1-SV40pA-Neo cassette of 6.8 kb was amplified by PCR. To obtain the targeting vector, these three DNA fragments were directionally subcloned into pMC1DTpA, using the In-Fusion Advantage cloning kit (Clontech, Mountain View, CA, USA). Homologous recombination in the ES cells and chimeric mice production were carried out as described previously. Resulting chimeric mice were crossed with C57BL/6 mice to establish the C2a-5FAD mice. Homozygous C2a-5FAD mice were obtained by crossing heterozygous pairs.

### Simulation

A multi-CPU system with cache coherent non-uniform memory access (NUMA) architecture (Altix 3000; SGI, Milpitas, CA, USA) having 256 processor cores (Intel Itanium 2, 1.5 GHz) and 512 GB globally-shared memory was utilized. The bandwidth of the NUMA interconnection was 6.4 GB/s. The system was equipped with SUSE Linux Enterprise Server 10, an optimized message passing toolkit (SGI MPT), Intel compilers (C and FORTRAN), and Math Kernel Library (MKL).

**Figure 7:**
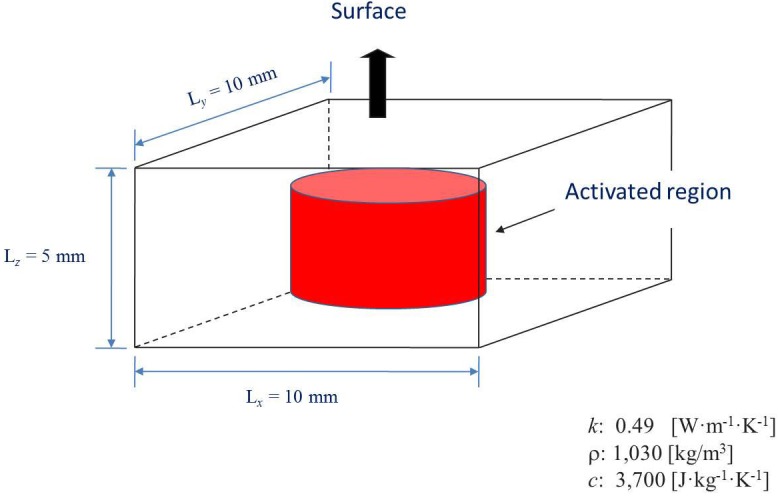
Simulation architecture.

Simulation architecture is shown in Figure 7. The governing equations were the Pennes Bio-Heat Transfer Equations as follows:

(equation 3) where T, is tissue temperature; k, tissue thermal conductivity; ρ, mass density; and c, heat capacity. Each term represents heat conduction, heat transfer to blood flow, and metabolic heat generation, respectively.

Mass flow rate of blood, Vf, in kg/m^3^/s is given with the following equation:

(equation 4) where CBF was given in mL/100 g/min.

Parameters were set based on known human PET data for the resting state with CBF: 55 mg/100 g/min; CMRglu: 6.1 mg/100 g/min (83% aerobic +17% anaerobic = 75 cal/100g/min); CMRO_2_: 3.8 mL/100 g/min. The ratios of increase associated with full activation is known to be CBF:CMRglu:CMRO2 = 4.1:4.1:1. Accordingly, the percent increase for each parameter is: CBF:34.5%; CMRglu:34.5% (8.41% aerobic +26.09% anaerobic = 9.86% aerobic equivalent), and CMRO_2_:8.41%.
